# The Amyloid-Beta Clearance: From Molecular Targets to Glial and Neural Cells

**DOI:** 10.3390/biom13020313

**Published:** 2023-02-07

**Authors:** Wenjun Cai, Tong Wu, Ning Chen

**Affiliations:** Tianjiu Research and Development Center for Exercise Nutrition and Foods, Hubei Key Laboratory of Exercise Training and Monitoring, College of Sports Medicine, Wuhan Sports University, Wuhan 430079, China

**Keywords:** Alzheimer’s disease, amyloid-beta, pluripotent stem cell, neuron, Aβ clearance

## Abstract

The deposition of amyloid-beta (Aβ) plaques in the brain is one of the primary pathological characteristics of Alzheimer’s disease (AD). It can take place 20–30 years before the onset of clinical symptoms. The imbalance between the production and the clearance of Aβ is one of the major causes of AD. Enhancing Aβ clearance at an early stage is an attractive preventive and therapeutic strategy of AD. Direct inhibition of Aβ production and aggregation using small molecules, peptides, and monoclonal antibody drugs has not yielded satisfactory efficacy in clinical trials for decades. Novel approaches are required to understand and combat Aβ deposition. Neurological dysfunction is a complex process that integrates the functions of different types of cells in the brain. The role of non-neurons in AD has not been fully elucidated. An in-depth understanding of the interactions between neurons and non-neurons can contribute to the elucidation of Aβ formation and the identification of effective drug targets. AD patient-derived pluripotent stem cells (PSCs) contain complete disease background information and have the potential to differentiate into various types of neurons and non-neurons in vitro, which may bring new insight into the treatment of AD. Here, we systematically review the latest studies on Aβ clearance and clarify the roles of cell interactions among microglia, astroglia and neurons in response to Aβ plaques, which will be beneficial to explore methods for reconstructing AD disease models using inducible PSCs (iPSCs) through cell differentiation techniques and validating the applications of models in understanding the formation of Aβ plaques. This review may provide the most promising directions of finding the clues for preventing and delaying the development of AD.

## 1. Introduction

### 1.1. Alzheimer’s Disease (AD) and Amyloid-Beta (Aβ)

AD is a progressive neurodegenerative disease characterized by the deposition of Aβ and the formation of neurofibrillary tangles with hyperphosphorylated Tau protein. In clinical practice, the patients present memory deficits, cognitive dysfunction, language and visual-spatial impairment, and behavioral dysfunction [1]. The global prevalence of AD is 20% and 5% among people over 85 and over 65 years old, respectively [2,3]. Early-onset AD (EOAD) and late-onset AD (LOAD) are the two subgroups of AD based on the age of onset. EOAD affects 5% of people, with symptoms appearing before 65 years old. The development of EOAD is associated with multiple genetic factors. The heritability of early AD is more than 90% [4]. Patients with familial AD carry mutations in either amyloid precursor protein (APP) or presenilin (PSEN1 or PSEN2) [5,6]. The mutation elevates the levels of Aβ monomers by altering the proteolytic way of APP. LOAD is the most prevalent type of AD. Extracellular Aβ deposition and neurofibrillary tangles caused by abnormal Tau protein hyperphosphorylation are usually observed in brain tissues of AD patients. Aβ alteration over time and space also contributes to the cognitive impairment of AD patients.

The amyloid cascade hypothesis was developed as a result of the discovery of the APP gene mutation, which is the first identified gene associated with AD [7]. Aβ is located in the transmembrane region of APP in neuronal synapses [8]. With the action of β-secretase in the ectodomain and γ-secretase in intra-membranous locations, APP can be hydrolyzed to produce a residue with 39–43 amino acids, known as an Aβ monomer. Aβ with a length of 42 amino acids is one of the most common monomeric fragments that are unstable and readily assemble into metastable oligomers through hydrophobic and hydrogen bonding interactions. Soluble oligomers are considered the most toxic form of Aβ. Oligomers are kinetic intermediates in fibrillar Aβ assembly reactions [9]. The assemblies of oligomers are transient [10]. Fibrillar Aβ is the major component of cerebral cortical plaques [11]. There are age-dependent Aβ isoforms present in the pathological brain, thus accelerating the formation of Aβ plaques [12]. A number of cell surface proteins have been reported to act as Aβ receptors. The receptors may trap Aβ oligomers and protofibrils on the neuronal surface and initiate the neurotoxic signals [13]. The factors including oxidative stress, imbalanced calcium homeostasis, dysfunctional autophagy, and Tau protein hyperphosphorylation can cause pathological changes in various brain cells [14]. Thus, Aβ-targeted therapeutic strategies are a promising approach for the treatment of AD.

### 1.2. Immunotherapy of Aβ Clearance

Targeting the primary toxic structures of Aβ to inhibit their aggregation is the most direct and effective way to alleviate neuronal damage. Immunotherapy includes active immunization and passive immunization [15]. Vaccines allow the body to actively produce antibodies. The first evidence of antibodies to resist cytotoxic aberrant amyloid is the one obtained from rabbits immunized with soluble toxic Aβ oligomers [16], indicating that a key requirement for AD vaccines is that the immunogen must be a toxic oligomer to elicit antibodies against the toxic conformational epitope. However, toxic oligomers induce strong pro-inflammatory responses that are detrimental to AD patients. The first Aβ immunotherapeutic candidate is AN1792 for targeting Aβ monomers at T-lymphocyte epitopes, which can efficiently reduce Aβ plaque and enhance cognitive capacity. In contrast, AN1792 also can cause hyperactivity of the innate and adaptive immune systems and induce highly harmful inflammatory responses. The clinical trial was terminated due to 6% of recruits experiencing an occurrence of meningitis [17,18]. Rationally designed AD vaccines can prevent the onset of this disease. Second-generation vaccines without T-lymphocyte epitopes are designed for targeting the N-terminal region and central domain of Aβ monomers. Peripheral administration of these antibodies against the N-terminal portion of Aβ can induce microglia-mediated phagocytosis, thereby ameliorating the Aβ-related pathology [19]. The new vaccines for targeting N-terminal of Aβ include Amilomotide and UB-311 [20,21].

Due to the limitations of active immunotherapy, passive immunotherapy strategies have also been trialed. Aβ plaques can be significantly reduced by exogenous injection of monoclonal antibodies targeting the brain. The drugs for targeting the N-terminal of Aβ as mentioned above also include Aducanumab, Donanemab and Lecanemab [20]. Aducanumab is the first Aβ-targeted monoclonal antibody drug approved by the FDA. The application of Aducanumab reduced the formation of Aβ plaques. However, the FDA mandates long-term safety monitoring of the drug, primarily involving the reduced risk of amyloid-related imaging abnormalities (ARIA). Clinical studies have documented that two-thirds of apolipoprotein E (APOE4) homozygotes are developed to ARIA during the treatment with Aducanumab. Prior to the decision for the application of Aducanumab, patients must be subjected to the verification of APOE genotypes because of the effect of APOE isoforms on ARIA risk. At present, most clinicians are still inexperienced with this novel treatment; thus, clinical investigators have developed appropriate use recommendations (AURs), which define patient selection criteria and MRI monitoring schedules [22], to assist in the correct application of Aducanumab in clinical practice. Recently, FDA has approved Lecanemab based on phase 2 trial data, as shown in a reduction in Aβ plaques and slow cognitive decline in patients at an early stage [23,24]. Aβ plaques that build up in the brain are ultimately toxic to certain brain cells. Gradual destruction of these brain cells results in the development of cognitive impairment [25].

The structure and modifications of Aβ play an important role in the production of monoclonal antibodies. Post-translational modifications of Aβ peptides accelerate their aggregation to induce toxicity, which in turn promotes the development of AD [26]. The isomerization of Asp residues of Aβ enhances its oligomerization, fibril formation and neurotoxic effect [27]. The monoclonal antibodies that recognize isoaspartate-modified Aβ can attenuate AD-like amyloid pathology in mice [28]. Previous studies have shown that the alanine to valine mutation of Aβ^A2V^ enhanced APP processing in homozygous individuals, whereas it has a protective effect in the heterozygous state [29,30]. Since N-terminal residues of Aβ are involved in fiber-to-fiber interactions, Aβ^A2V^ heterozygotes perturb hydrogen bonding and inter-sheet organization, thereby preventing fibrillary Aβ generation. According to this method, an all-D-isomer synthetic peptide is designed to limit the first six amino acids of the N-terminal sequence of A2V-mutated Aβ and can successfully inhibit the nucleation of Aβ by intranasal delivery to APPSwe/PSEN1dE9 mice. Furthermore, long-term treatment with Aβ^A2V^ peptide can reduce synaptic loss caused by Aβ oligomerization in transgenic mice, even in the brain region distant from the olfactory bulb [31]. In addition to Aβ antibodies, microglia-based immunotherapies through targeting receptor expressed on myeloid cells 2 (TREM2), CD38 and Toll-like receptors (TLRs) are also in development [20,32]. With the advances in single-cell RNA sequencing and spatial transcriptome sequencing, more promising molecules for clearing Aβ by exogenous administration into the brain will be discovered for passive immunotherapy.

Aβ deposition-induced oxidative stress and inflammatory responses are the major reason for Tau pathology in AD. Glycogen synthase kinase-3 beta (GSK-3β) is a key enzyme for Tau protein hyperphosphorylation. GSK-3β is involved in the signaling pathways such as mammalian target of rapamycin (mTOR) [33], spleen tyrosine kinase (SYK) [34], phosphoinositide 3-kinase (PI3K) and protein kinase B (AKT) [35]. Inhibiting the activity of GSK-3β is an option in the quest of AD therapy. The GSK-3β inhibitor lithium improves spatial learning and memory capacity in AD animal model [36]. Another inhibitor, melatonin, also can modulate GSK-3β level and reduce Aβ production [37].

### 1.3. The Evolution of the AD Hypothesis: From Cascades to Interaction

The amyloid cascade hypothesis is a neuron-centric linear model. According to this hypothesis, Aβ deposition outside neurons can initiate Tau fibrillar tangles, oxidative stress, synaptic dysfunction, inflammatory reactions and ultimately neuronal death. Aβ deposition occurs 20–30 years before amnesia and neuronal loss [38,39]. The genetic data from lab animals and clinical samples support Aβ deposition as an upstream event over the past 25 years. Aβ plaque is now accepted to be a protective mechanism, rather than the cause of AD [40].Many studies have brought doubt into this linear model [41]. The first question is the relationship between Aβ deposition and Tau fibrillary tangles. According to one of the previous studies, Aβ deposition occurs before the formation of Tau fibrillary tangles, which is assumed to be responsible for the pathological accumulation of Tau and Tau-mediated neurodegeneration [42]. Recently, the spatiotemporal development of Aβ and Tau-pathology in AD patients has been confirmed by neurofunctional imaging using Positron Emission Tomography (PET), indicating the initial formation of Aβ in the cortex, a part of the brain with high metabolic demand, and the spreading from the neocortex to the brainstem, and the cerebellum finally [43,44]. The occurrence of Tau fibrillar tangles is later than Aβ deposition. Tau pathology is initially apparent in the endothelial cortex, and then spreads to the limbic regions and eventually the neocortex [45,46,47]. These studies reveal differences in the temporal and spatial distribution of Aβ plaques and Tau proteins. In addition, the progression of Aβ stress and Tau pathology is complex and indirect at the molecular level [48]. Compared with Aβ plaques, Tau pathology has a closer relationship with the loss of neurons as the extension of both time and space. Tau pathology may develop independently from Aβ deposition. Over the decade, the amyloid cascade hypothesis has developed from a linear toxicological model to an integrated model influenced by multiple disease mechanisms.

Recent studies have highlighted the significance of active astrocytes and microglia surrounding Aβ deposits [49]. In the earliest description of AD, the formation of Aβ plaques, microglial aggregation, astrogliosis and neuronal dystrophy are reported as the hallmarks of AD. One study has applied scRNA-seq to extensively characterize the transcriptional responses of 13 cell types associated with Aβ deposition in brain tissues of AD mouse models to explore the diverse transcriptional responses from microglia, astrocytes and oligodendrocytes, indicating that the interaction across different cell types may affect the onset and progression of AD [50]. One study has resolved complex three-dimensional (3D) cellular anatomy of fixed human brain samples in tissue repositories, thus uncovering the special 3D structures of neurons, microglia cells and astrocytes in AD, and the complex interaction of these cells responding to Aβ plaques [51].

## 2. Clearance of Aβ by Glial Cells

### 2.1. Microglia and Aβ Clearance

**Figure 1:**
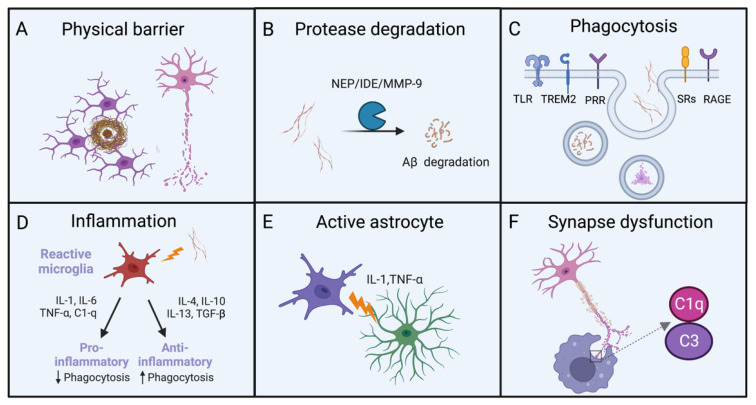
Clearance of Aβ by glial cells. (**A**) Microglia form a physical barrier to protect neurons. (**B**) Increasing the breakdown by microglial proteases could enhance the clearance of Aβ deposits. (**C**) The phagocytic activity of microglia could be enhanced by targeting receptors and signal pathways involved in this response. (**D**) Inflammation caused by microglia produce both pro-inflammatory and anti-inflammatory factors. (**E**) Microglia can secrete cytokines to activate astrocytes. (**F**) Microglia participate in amyloid-dependent synaptic dysfunction.

Microglia are innate immune cells in the brain responsible for pathological changes by continuously monitoring their microenvironment (Figure 1). Aβ deposition can induce the migration and proliferation of microglia. Microglia surrounded and compacted Aβ plaques can form a physical barrier to prevent the generation of new Aβ monomers and execute the protection of neurons [52]. Insufficient clearance of Aβ has been identified as the major pathological mechanism of AD. Microglia also can promote the breakdown of Aβ fibrils and oligomers by secreting proteinase such as insulin-degrading enzyme (IDE), epinephrine (NEP) and metalloprotease-9 (MMP-9) [53]. Besides degrading extracellular Aβ fibrils and oligomers, microglia can express a series of receptors to enhance the uptake of Aβ aggregates. When pattern recognition receptors (PRRs), TLRs, scavenger receptors, receptor for advanced glycation end-products (RAGE), TREM2 and other receptors are activated, the uptake of Aβ by microglia is significantly increased [54,55]. Researchers have used radioisotopes or fluorescent dyes to identify Aβ before injecting them into rats. Microglia have been found to remove Aβ by surface receptor-mediated phagocytosis, as confirmed by the detected pro-inflammatory cytokines [56,57]. Extracellular ligand binding can activate a cascade of intracellular signaling events that lead to phagocytosis and inflammation.

Aβ deposition is accompanied by the activation of the innate immune system. The microglia surrounding Aβ plaques with altered morphology and activation state have higher levels of inflammatory factors [58,59]. In the early stage of AD, activated microglia prevent the accumulation of toxic Aβ by phagocytosis and achieve the protection of neurons. During the extended progression of AD, overexpressed pro-inflammatory cytokines can initiate the impaired function of phagocytosis and result in the accelerated Aβ deposition [60,61]. Conversely, the up-regulated anti-inflammatory cytokines enhance the phagocytotic function of glial cells. These studies have also demonstrated that anti-inflammatory cytokines such as IL-4 and IL-10 inhibit prostaglandin E2 (PGE2) and corresponding signaling pathway, thereby preventing pro-inflammatory cytokines from inhibiting Aβ-induced phagocytosis, which highlights the roles of phagocytosis and inflammatory responses in the clearance of Aβ. By interfering with receptor-ligand interactions or inhibiting their downstream signaling pathways, microglia can further effectively remove Aβ.

During the developmental progress of the brain, microglia can clean redundant synapses to strengthen neuronal circuits, while Aβ deposition can induce a transition of microglia from a resting state to a reactive state. Hyperactivated microglia impair synaptic function, and impede cell communication, growth and development. Activated microglia promote astrocyte activation through the released inflammatory factors for inducing toxic and harmful Aβ to damage neurons. A recent study using mouse AD models has demonstrated that astrocytes and microglia can contact and clear synapses through the C1q complement pathway, thus resulting in the exacerbated progression and severity of AD [62]. On the other hand, C1q deletion can execute a neuroprotective effect in AD model mice, suggesting that the inhibition of C1q complement pathway is an attractive strategy to delay neurodegenerative diseases including AD, through regulating the elimination of complement C1q-dependent excitatory and inhibitory synapses by astrocytes and microglia. Likewise, another study has revealed that the glucagon-like peptide-1 receptor (GLP-1R) agonist NLY01 selectively blocks Aβ-induced microglial activation and inhibits the differentiation of reactive astrocytes. NLY01 acts directly on GLP-1R-positive microglia and improves spatial learning and memory capacity in transgenic AD mouse models [63].

Transplantation of Aβ from transgenic host tissue into non-transgenic host tissue can result in the invasion and deposition of Aβ in the non-transgenic host tissue, thereby inducing neurodegeneration [64]. The experimental data show that the isomerization of Aβ can trigger the aggregation and propagation of Aβ [12]. However, the precision mechanism of Aβ propagation into wide-type (WT) grafts is unknown. Recent studies suggest that Aβ entry into WT grafts is accompanied by microglial infiltration. Regulating microglial function may reduce Aβ deposition in WT grafts [65]. Aβ seeding and diffusion are associated with inflammasome activity and accompanied by the formation of inflammatory vesicle-dependent apoptosis-associated speck-like protein containing CARD domain (ASC) specks. ASC released from microglia can rapidly bind to Aβ and stimulate the production of Aβ oligomers and aggregates, thereby exacerbating the progression of AD [66].

### 2.2. Targets of the Aβ Clearance by Microglia

APOE is the major risk gene of LOAD. APOE4 variants are present in 14% of the population and in 50% of AD patients [67]. Compared to those with normal APOE, people with one copy of APOE4 have three times higher risk of developing AD, and the risk is increased by 15 times for those with two copies of APOE4 [68]. APOE has three alleles: APOE2, APOE3 and APOE4, with the difference in amino acid residues at positions 112 and 158, thereby leading to structural and functional differences [69]. The isoform APOE2 is thought to be protective against AD. A person who has APOE3 is at the same risk of developing AD as those with one copy of APOE4 [70]. APOE can affect the clearance of soluble Aβ monomers in an isomer-dependent pathway: APOE4 > APOE3 > APOE2 [71,72,73]. Studies have shown that targeting APOE4 can reduce Aβ pathology. Preventing APOE4 interaction with Aβ by small molecules, peptides, antibodies or antisense oligonucleotides can remove 60% of APOE mRNA and protein from the brain [74]. APOE regulates lipid homeostasis by mediating lipid migration in cells [75]. ATP-binding cassette transporter A1 (ABCA1) and ABCG1 receptors on the cell surface can transfer cholesterol and phospholipids to the developing APOE for the formation of lipoprotein particles after APOE is released. APOE binds to receptors on the cell surface and then redistributes lipids and cholesterol into neurons [76]. However, APOE binds to aggregated Aβ and other types of Aβ in the extracellular space when Aβ is present, which may impair the communication capability to neighbor neurons as excessive lipids build up. Extra lipid molecules may attach to potassium channel protein in the cell membrane of neurons, which can prevent neurons from firing [77], thereby resulting in reduced neuronal excitability during the progression of AD. Long-term Aβ and lipid aggregates could induce inflammatory reactions. Targeting APOE may help to recover lipid metabolism in microglia.

It was found that interference with lipid droplet formation by a drug called Triacsin C could reverse lipid overload in microglia with APOE4. Normal communication between microglia and neighboring neurons is restored [78]. Lipid homeostasis is critical for different types of cells in brains of AD patients; therefore, it is not a problem limited to microglial cells. Restoring lipid homeostasis in multiple cell types would be an important direction to slow down the progression of AD.

### 2.3. New Target on Microglia: TREM2

**Figure 2:**
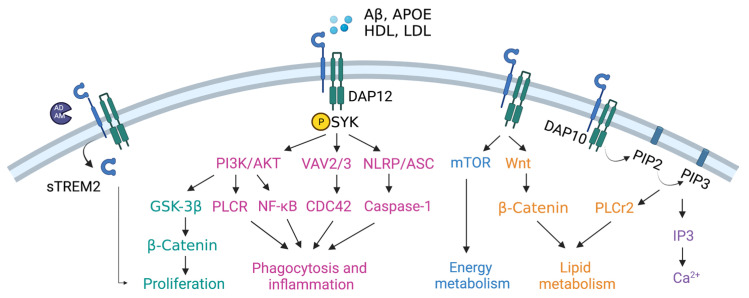
The cascade signaling pathways are mediated by TREM2 in microglia. sTREM2 can promote microglial proliferation. TREM2 can also promote microglial proliferation by activating the GSK-3β/β-catenin signal pathway. Meanwhile, TREM2 can promote phagocytosis and inflammatory response through the PI3K/AKT-PLCR, PI3K/AKT-NF-κB, VAV2/3-CDC42 and NLRP-caspase-1 signal pathways. TREM2 can regulate energy metabolism of microglia through mTOR signal pathway, and lipid metabolism through Wnt-β-catenin and PIP3-PLCr2 signal pathways. TREM2 also can affect calcium homeostasis through regulating the PIP2/3-IP3 signal pathway.

TREM2 has been identified as a novel risk gene in AD with significant impacts on Aβ deposition [79,80]. The association between TREM2 and AD is second to APOE4. Studies have documented that TREM2 with R47H mutation can increase the risk of LOAD by three to five times [81,82]. TREM2 is a member of the immunoglobulin (Ig) receptor superfamily and is expressed on microglia in the brain tissue. The interaction between TREM2 and Aβ is mediated by microglia. When microglia are suppressed in 5xFAD mice using the receptor of the colony-stimulating factor-1 (CSF-1R) inhibitor PLX3397, TREM2 injection does not alter the content of Aβ [83]. TREM2 affects the function of microglia from the aspects of proliferation, phagocytosis, inflammation, energy metabolism, lipids metabolism and calcium homeostasis (Figure 2).

TREM2 is a single transmembrane protein receptor composed of three domains. The extracellular domain could be cleaved and released by enzymes from the A Disintegrin and Metalloprotease (ADAM) family to form soluble TREM2 (sTREM2), with protective roles in microglial survival and propagating proinflammatory signals. The extracellular domain could bind to high-density lipoprotein (HDL), low-density lipoprotein (LDL), APOE and Aβ, to activate TREM2 and in turn activate the transmembrane domain DNAX-activating protein of 12 kDa (DAP12). DAP12 contains an immunoreceptor tyrosine-based activation motif (ITAM), and can recruit SYK to activate downstream molecules, such as VAV, proline-rich tyrosine kinase 2 (Pyk2) and nucleotide-binding oligomerization domain, leucine rich repeat and pyrin domain containing protein (NLRP). Another transmembrane domain is DAP10, which can activate PI3K and inositol 1,4,5-trisphosphate (IP3) and further change calcium concentrations in microglia [84,85].

TREM2 is crucial in the proliferation and migration of microglia toward Aβ plaques. Microglia exhibit migration defects in the TREM2 R47H knockout mouse model with fewer microglial aggregates surrounding Aβ plaques [86]. Microglia with overexpressed TREM2 are more likely to ingest amyloids, whereas those lacking TREM2 have lower levels of phagocytes and activated microglia in AD mouse models [87]. TREM2 can initiate phagocytosis and inflammatory response through SYK-PI3K/AKT, SYK-VAV2/3-CDC42 and SYK-NLRP-caspase-1 signal pathways [88,89,90,91,92]. Increasing evidence shows aberrant inflammasomes in AD brain. Due to the complex relationship between pro-inflammatory and anti-inflammatory responses, it is still not clear how TREM2 is involved in the inflammatory response, pyroptosis and phagocytosis in AD. The effect of inflammation on TREM2 expression shows opposite results in vitro and in vivo. Chronic inflammation can up-regulate TREM2 under pathological conditions with AD, and acute inflammatory responses can reduce TREM2 generation in vitro models [93,94]. In either case, TREM2 supports the activity of the microglia [95].

TREM2 has also been identified as a lipid sensor in microglia, with the enrichment of the genes for regulating cholesterol biosynthesis and other lipid biosynthesis pathways. The extracellular domain of TREM2 can bind lipids, apolipoprotein and cell debris, thereby affecting the metabolism of cholesterol, myelin and phospholipids in microglia [84,96].

Studies have revealed that HDL-mediated lipid transport is restricted in TREM2 mutant microglia, as well as the expression of lysosomal genes. Researchers have transplanted wild-type TREM2 and heterozygous TREM2-R47H into a chimeric AD mouse model and found that microglia with TREM2-R47H mutant have lower uptake of Aβ-lipoprotein complexes and more sensitivity to the reduction of Aβ plaques [97]. TREM2 also can mediate lipid metabolism through the Wnt-β-catenin and PLCr2 signal pathways [89]. The energy metabolism is regulated by the mTOR signal pathway [98].

### 2.4. Astrocytes and Aβ Clearance

In the central nervous system, astrocytes regulate the function of the synapse between neurons, maintain the homeostasis of neurotransmitters and ions, and provide nutrients for neuronal metabolism [99]. Astrocytes are in close proximity to neurons and respond to signals from those neurons. With the analysis of postmortem samples from AD patients, an increase in astrocytes around Aβ plaques is observed [51]. Aβ aggregates can stimulate the production of chemical molecules, such as monocyte chemotactic protein 1 (MCP-1), to recruit astrocytes [100]. Aβ-induced astrocytes undergo morphological, molecular and functional alterations. In response to pathogenic stimuli, astrocytes exhibit a variety of reactive phenotypes for overexpressing particular proteins. Identification of disease-associated reactive astrocytes can be aided by single cell sequencing. The transcriptome investigation in AD model mice has identified multiple molecular phenotypes of the reactive astrocytes [101,102]. In the cerebrospinal fluid of AD patients, glial fibrillary acidic protein (GFAP)-positive astrocytes are found to be related to elevated Aβ levels, whereas YKL-40, also known as chitinase-3-like protein 1 (CHI3L1)-positive astrocytes, are found in association with elevated Tau levels. GFAP mostly serves as a structural protein, while YKL-40 is believed to play a direct role in the inflammatory response by activating the innate immune system. The role of astrocytes in AD may be better understood by targeting specific types of reactive astrocytes [103,104].

Activation of astrocytes is mediated by microglia. Microglia release cytokines such as interleukin-1α (IL-1α), IL-1β, IL-6, tumor necrosis factor alpha (TNF-α), and the complement component C1q to induce the generation of activated astrocytes [105]. Reactive astrocytes are involved in Aβ clearance in vitro, through multiple receptors, including RAGE receptors, LRP receptors, membrane-associated proteoglycans and scavenger receptors, to execute the recognition and uptake of Aβ [106,107]. Aβ induces multiple downstream signaling cascades in astrocytes including Janus kinase (JAK)-signal transducer and activator of transcription 3 (STAT3), nuclear factor-κB (NF-κB) and calcineurin-NFAT (nuclear factor of activated T cells) signal pathways [108,109]. Among them, the JAK2-STAT3 signal pathway is emerging as a central regulator to maintain astrocyte reactivity, as shown in the studies from both rat and non-human primate models [108,110].

Active astrocytes seriously affect calcium homeostasis and its interaction with synapses [111,112]. Disturbance of calcium homeostasis may adversely affect synaptic function. Approximately 80% of glutamate uptake and restoration in the synaptic interval space is carried out by the glutamate transporter-1 (GLT-1) and glutamate and aspartate transporter (GLAST) on astrocytes. Downregulated GLT-1 is a common feature of AD, which might alter extracellular glutamate levels, and result in excitotoxicity and neurodegeneration [113].

In addition, the production of active astrocytes is accompanied by the release of excessive gamma aminobutyric acid (GABA) and the activation of neuronal GABA receptors, thereby suppressing the pro-inflammatory response of astrocytes. Astrocytic GABA-mediated neuroprotection might be a common feature of reactive astrocytes [114].

As the major expression vector of APOE, astrocytes are able to regulate Aβ accumulation through cholesterol signaling. Super-resolution imaging in the mouse brain shows that APOE utilizes astrocyte-derived cholesterol to specifically transport neuronal APP into and out of lipid clusters, and when cholesterol synthesis in astrocytes is reduced, APP effluent lipid clusters that interact with α-secretase to generate soluble APP-α (sAPP-α) potently reduce Aβ and Tau burden in AD mouse models [115].

## 3. Experimental Models of Aβ Clearance

### 3.1. Defects in Traditional Animal Models and Advances in iPSC Models

Since AD is characterized by a systemic degenerative change in the brain, which has great limitations on the studies of AD patients, animal replacement models of AD are indispensable in clinical studies. Numerous strategies with obvious efficacy in AD rodent models have failed in clinical trials. Species variability hinders the translation of clinical drug studies. Genetic modification in rodent models only slightly alters their pathology and behavior [116]. Rodent models can not accurately capture pathological disorders and cognitive impairment of real AD patients. The generation of Aβ from modified genes in rodent models is an acute process, while Aβ deposition observed in AD patients is a chronic process that steadily becomes worse over time [117]. Upon acute stimuli, Aβ can trigger metabolic reprogramming of microglia from oxidative phosphorylation (OXPHOS) to glycolysis, depending on the mTOR-HIF-1a signal pathway. Chronic exposure to Aβ can induce metabolic defects of the microglia [118]. The distinctions between acute and chronic pathophysiology are examined in animal models and human samples. The results have demonstrated the difference in terms of gene expression, regulation and functions. At the single-cell level, animal models and AD patients have different cellular compositions. In three neuronal subtypes, two oligodendrocyte subtypes, microglia, astrocytes, oligodendrocyte progenitor cells and endothelial cells, the gene expression profiles of AD patients differ from those of mice. Microglia are the cells in response to Aβ with the greatest variety. There is little overlap for gene expression profiles in microglia of AD patients and mouse models, except for APOE. In addition, human glial cells differ from animal glial cells in number, structure and function. According to one previous study, activated microglia in the hippocampus of AD patients are quantitatively different from those in the APP mouse model [119]. Human glial cells are 2.6 times larger in diameter and 16.5 times larger than rodent glial cells. The calcium signals spread four times faster in human glial cells. As a result, cytosolic calcium can reveal a dramatic increase in response to metabotropic glutamate receptor agonists [120].

Given the difference between species, animal models cannot fully present the characteristics and functions of AD patients. It is critical to explore Aβ pathology in accurate cell models with human diseases [118,121,122,123]. Based on the continuous development of stem cell models, increasing studies are conducted on iPSCs to explore the precision mechanisms of AD [124].

The establishment of fibroblast-derived iPSCs provides new possibility for exploring AD pathology [125]. There are three types of AD cell models generated from iPSCs. The first is using normal iPSCs to develop an AD phenotype through the induction of chemical inducers such as aftin5. This model has neurotoxicity and limited formation of Aβ plaques [126]. The second is to differentiate iPSCs into neurons from AD patients. These iPSCs often carry mutations in one or multiple genes, such as PS1, PS2, APP, and APOE [112,127]. The iPSCs derived from this method display Aβ deposition: a pathological hallmark of AD. The third is to apply Cas9-mediated gene editing technology to induce the overexpression and silence of AD-associated genes in iPSCs [128].

Cell models using patient-derived iPSCs that can be differentiated into neurons, microglia and astrocytes are different from animal models, and contain complete disease background information with the capability to replicate disease progression in vitro [53,129,130]. The iPSCs from AD patients have the capacity to differentiate into different subtypes of neurons and non-neurons, thus providing a useful strategy for observing and investigating phenotypes related to AD [128]. In a study using iPSCs produced from patients with hereditary APP mutations and spontaneous variants, elevated amounts of Aβ and phosphorylated Tau are determined. The phosphorylation of Tau in human neurons is involved in the proteolytic process of APP [131]. The iPSC models also reveal that the accumulation of hyperphosphorylated Tau is triggered by Tau oligomers, but not monomers [132]. Single-cell sequencing data from AD patients indicate that neurons culturing with or without microglia could result in different gene expression patterns. Microglia cultivated alone exhibit more expression of APOE [128]. An automated culture platform for iPSC-derived neurons, astrocytes and microglia is established to improve cell models and achieve cellular phenotypes for proliferation, migration, phagocytosis and neurotrophic signaling transmission [133].

### 3.2. The Advances in a Co-Culture Cell Model

In vitro cell culture of neurons and non-neurons that comprehensively recapitulates cell-to-cell communication provides an ideal model for exploring the pathology of AD. Neurons can reveal the increased post-synaptic marker expression and a higher number of branches and junctions when co-cultured with glial cells. With evaluation and comparison of the transcriptome, the up-regulated genes involved in mature neuronal process and synchronized neural network activity are usually observed in the co-culture system [134]. Microglia display a less inflammatory phenotype when co-cultured with neurons and astrocytes. In detail, transforming growth factor beta 1 (TGF-β1) as the anti-inflammatory marker is increased and IL-1β as the pro-inflammatory marker is decreased [135]. The intracellular deposits of Aβ are significantly reduced in the co-culture system [136]. The mechanism of microglia for the removal of Aβ plaques is explored. The result indicates that the addition of microglia to the co-culture system can provide approximately 25% basal protection of neuronal health and increase the formation of Aβ plaques by three times, suggesting that Aβ plaque formation and compaction may be neuroprotective in a feedback manner. The addition of pro-inflammatory cytokines and Aβ42 oligomers to the co-culture system can increase Aβ plaque formation by six folds, but neuroprotection is lost. The overactivation of microglia by pro-inflammatory factors counteracts neuronal protection [135]. The protective role of glial cells on neurons is also reflected in the regulation of glutamate-induced excitotoxicity. The co-culture system significantly reduced neuron loss and astrocyte hypertrophy [137]. New findings suggest that Piezo type mechanosensitive ion channel component 1 (PIEZO1) receptors drive Aβ clearance in microglia [138]. The co-culture system of neurons, astrocytes and microglia more accurately simulates the in vivo response to Aβ stimulation, mechanical injury and glutamate-induced excitotoxicity. Clarifying the roles of microglia, astrocytes and neurons in Aβ plaque formation using co-culture systems may lead to the discovery of new targets for preventing AD development.

### 3.3. Crosstalk between Neurons and Non-Neurons

**Figure 3:**
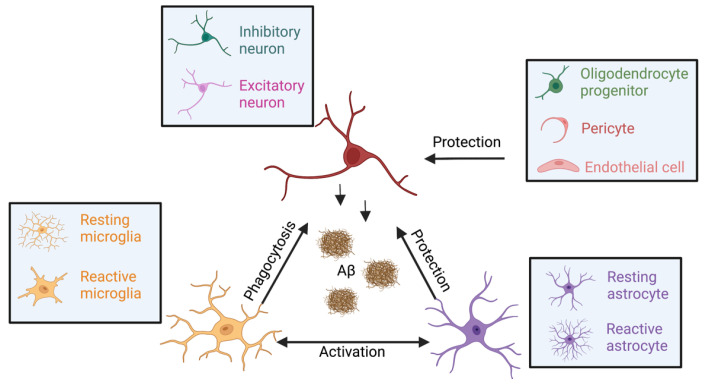
The crosstalk between neurons and non-neurons.

Aβ activates the function of multiple different cell types of the brain (Figure 3). Microglia is the innate immune cell in the brain that mainly influences the neurons by phagocytosis and synaptic pruning. Microglia can respond more rapidly than astrocytes to pathological stimuli during the occurrence of Aβ, and induce the activation of astrocytes, as well as determine their fate [139]. Activated microglia can also continuously monitor their microenvironment and respond to pathological changes [140]. Negative feedback regulation of neuronal activity by microglia is critical for neural protection. Microglia can inhibit neuronal activity by sensing and breaking down extracellular ATP released from neurons upon activation. Astrocytes communicate with microglia by releasing glutamate, calcium ions and ATP to activate various microglial purinergic receptors. Activated microglia resolve neuronal insults through phagocytosis, cytokine release and cellular repairing [141]. In response to Aβ, astrocytes can produce the complement factor C3 that binds to C3a receptor in neurons and affects synaptic architecture and cognitive function [142]. In addition, astrocytes can compensate for microglial phagocytosis in pathological situations. The number of excitatory synapses phagocytosed by microglia in AD model mice is significantly reduced after the knockout of TREM2, but can not affect the function of excitatory synapses phagocytosed by astrocytes. Moreover, the knockout of TREM2 can promote astrocyte phagocytosis of inhibitory synapses in AD model mice. Synergistic effects between microglia and astrocytes are protective for neurons. The primary role of oligodendrocytes is the generation of the myelin sheath that supports fast excitatory and inhibitory synaptic transmission. Pericytes, together with astrocytes and vascular endothelial cells, build up the blood–brain barrier that impedes the molecules out of the central nervous system.

During the past decade, studies have focused on cellular effects at the level of broad cell types and comparatively less focus has been placed on subtypes of neurons [143]. Recent advances in high-throughput, large-scale profiling of AD pathological samples allow for the identification of specific cell types associated with the disease [144,145]. The major cell types include excitatory neurons (marked by NRGN), inhibitory neurons (GAD1), astrocytes (AOP4), oligodendrocytes (MBP), microglia (CSF1R and CD74), oligodendrocyte progenitor cells (VCAN), endothelial cells (FLT1) and pericytes (AMBP) [146]. Based on the specific marker gene expression of subtype cells, the relative proportions are low for most neuron types and high for non-neuron types. Specifically, excitatory neurons are detected to be reduced in AD patients [147]. The single-cell RNA sequencing study has identified an AD-associated excitatory neuron population (C3:Ex.Neuron) marked by netrin G1 (Ntng1), with down-regulated AD signal pathways at an early stage of AD [148]. Microglia have been shown to inhibit neural activity in response to glutamate-induced activation, and microglia work in a similar manner to inhibitory neurons, using negative feedback mechanisms that are critical for neural protection [140]. The impact of AD pathology on inhibitory neurons is under-explored. A study shows the GABA antagonist bicuculline (BIC) has a greater influence on bursting activity in co-culture versus simple neuronal cultures. This is because most GABA receptors are expressed in astrocytes and oligodendrocytes, and BIC elicits the release of gliotransmitters such as glutamate, cytokines and GABA to regulate downstream neuronal activities [134]. The functions and contribution of these types of non-neurons to AD remain incompletely understood.

The essence of aphasia, amnesia and dysfunction is the damage to the neural circuitry and an imbalance in the transmission of neurotransmitters, ionic signals and energy in cells. There remain many unsolved questions about how the interaction between neurons and non-neurons is dysregulated in diseases. Understanding cellular interactions may be central to clarify the pathogenesis of AD [149].

## 4. Future Remarks and Prospects

### 4.1. Pluripotent Stem Cell-Differentiated Neuronal Models

The application of iPSC-differentiated neuronal models has become an attractive strategy to study neurodegenerative diseases. Current strategies using small molecules and transcription factors to differentiate iPSCs into neural stem cells, glial cells and cerebral organoids have been proposed [150]. In future studies, there is a real demand to continuously expand the subtypes of neurons that can be generated and simulate the complex cultural environment of different types of cells in vitro. More efforts are needed to improve the purity and maturity of differentiated cells and standardize differentiation methods for achieving repeatable results.

### 4.2. 3D Cell Models

Improvement in 3D brain organoid culture could open a novel avenue to explore the pathogenesis of AD. The 3D culture of organoid brain could promote neuronal and glial differentiation when compared with 2D culture. It can accurately reflect the complex interactions of neural cells. The 3D models composed of neurons, astrocytes and microglia reflect microglial recruitment, neurotoxic activity such as axonal cleavage, and nitrogen monoxide release damage. Aβ in the 3D culture system exhibits higher-level production and more diffusion of Aβ into culture media. The absence of an interstitial compartment is believed to inhibit extracellular Aβ deposition in the 2D culture system [151]. New technology and multicellular models promise to reveal a clearer understanding of cellular and molecular mechanisms in response to Aβ.

### 4.3. Aging Characteristics

Limited access to human brain tissue has hampered the progress in studies on various age-associated neurodegenerative diseases at the cellular and molecular levels. The rejuvenation effect of iPSC reprogramming is a major drawback in modeling age-related diseases. In the past years, researchers have used various strategies to uncover aging characteristics in the iPSC models, such as inhibiting telomerase activity, creating DNA damage and editing the genome to human iPSCs [152]. In order to overcome the loss of age- and environment-related epigenetic signatures during the iPSC reprogramming, the directly differentiated fibroblasts have also been co-cultured with neurons (Fib-iNs) to explore neurodegenerative diseases [153]. Fib-iNs preserve many signatures of aging phenotypes, including transcriptomic, epigenetic, nuclear morphological, mitochondrial and other aspects of cellular senescence, which makes them appropriate for studies on AD in vitro [154].

### 4.4. AD-Related iPSC Bank

To better utilize iPSCs to elucidate neurodegenerative diseases, the researchers are planning to obtain iPSCs in healthy donor cells and have constructed 134 variants associated with AD. After differentiating the cells into neurons and glia, their morphology, transcriptomics and proteomics will be characterized, and the data will then be stored in open repositories [155]. By creating a series of heterologous cell lines from the same starting cells, scholars will be able to study all mutations in the same genetic background. Establishing standardized cell lines will be helpful for increasing the reproducibility of studies and conducting the comparison among different studies, which will greatly improve the understanding of AD pathology.

## 5. Summaries

**Figure 4:**
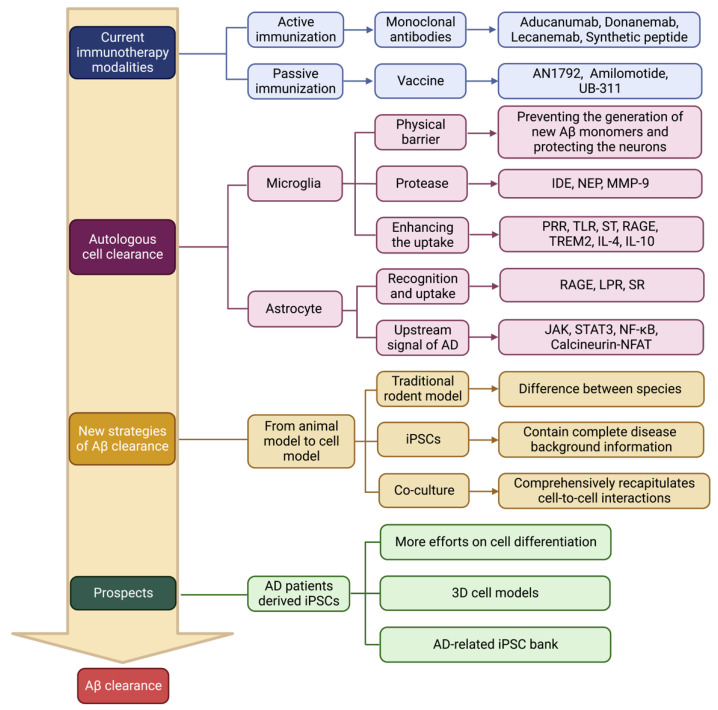
The summary of Aβ clearance from drugs or cell models in current studies.

This article summarizes the strategies of Aβ clearance during AD therapy in recent years and the establishment of novel AD models for understanding Aβ clearance (Figure 4). Following unsuccessful clinical application of Aβ studies, the recent focus has shifted to a more comprehensive perspective. Exploring the roles of glial and neural cells in the clearance of Aβ and the factors involving these processes should have great prospects. Changes in a variety of synergistic interactions ultimately lead to neuronal death in the brain. The underlying mechanisms are still largely unknown. Simple linear toxicity models are no longer suitable for explaining the pathological changes of AD. The accumulation and aggregation of Aβ is the initiator of pathological changes, but inhibiting Aβ accumulation is not a critical component during the treatment of neurodegenerative diseases. Understanding the roles of microglia and astrocytes in disease networks may be required to prevent the cascades induced by accumulated Aβ. Combining different types of cells in the brain to treat multiple nodes is highly necessary because the complex mechanisms of neurodegenerative diseases are involved in the interactions of multiple cells including both neurons and non-neurons. AD patient-derived iPSCs carrying accurate disease information can differentiate into diverse types of cells in brain tissues and can provide a promising strategy for uncovering neuronal interactions.

## Data Availability

Not applicable.
